# Field and experimental evidence of a new caiman trypanosome species closely phylogenetically related to fish trypanosomes and transmitted by leeches

**DOI:** 10.1016/j.ijppaw.2015.10.005

**Published:** 2015-10-21

**Authors:** Bruno R. Fermino, Fernando Paiva, Priscilla Soares, Luiz Eduardo R. Tavares, Laerte B. Viola, Robson C. Ferreira, Robinson Botero-Arias, Cátia D. de-Paula, Marta Campaner, Carmen S.A. Takata, Marta M.G. Teixeira, Erney P. Camargo

**Affiliations:** a Departamento de Parasitologia, Instituto de Ciências Biomédicas, Universidade de São Paulo, São Paulo, Brazil; b Centro de Ciências Biológicas e da Saúde, Universidade Federal do Mato Grosso do Sul, Campo Grande, Mato Grosso do Sul, Brazil; c Caiman Research in Conservation and Management Program, Instituto Mamirauá para o Desenvolvimento Sustentável, Tefé, Amazonas, Brazil; d Faculdade de Agronomia e Medicina Veterinária, Universidade de Brasília, Brasília, DF, Brazil

**Keywords:** Crocodilian, *Trypanosoma*, Reptilian parasites, Phylogeny, Phylogeography, Leeches, Co-infection, South America

## Abstract

*Trypanosoma terena* and *Trypanosoma ralphi* are known species of the South American crocodilians *Caiman crocodilus, Caiman yacare* and *Melanosuchus niger* and are phylogenetically related to the tsetse-transmitted *Trypanosoma grayi* of the African *Crocodylus niloticus.* These trypanosomes form the Crocodilian clade of the terrestrial clade of the genus *Trypanosoma.* A PCR-survey for trypanosomes in caiman blood samples and in leeches taken from caimans revealed unknown trypanosome diversity and frequent mixed infections. Phylogenies based on SSU (small subunit) of rRNA and gGAPDH (glycosomal Glyceraldehyde Phosphate Dehydrogenase) gene sequences revealed a new trypanosome species clustering with *T. terena* and *T. ralphi* in the crocodilian clade and an additional new species nesting in the distant Aquatic clade of trypanosomes, which is herein named *Trypanosoma clandestinus* n. sp. This new species was found in *Caiman yacare, Caiman crocodilus* and *M. niger* from the Pantanal and Amazonian biomes in Brazil. Large numbers of dividing epimastigotes and unique thin and long trypomastigotes were found in the guts of leeches (*Haementeria* sp.) removed from the mouths of caimans. The trypanosomes recovered from the leeches had sequences identical to those of *T. clandestinus* of caiman blood samples. Experimental infestation of young caimans (*Caiman yacare*) with infected leeches resulted in long-lasting *T. clandestinus* infections that permitted us to delineate its life cycle. In contrast to *T. terena, T. ralphi* and *T. grayi,* which are detectable by hemoculturing, microscopy and standard PCR of caiman blood, *T. clandestinus* passes undetected by these methods due to very low parasitemia and could be detected solely by the more sensitive nested PCR method. *T. clandestinus* n. sp. is the first crocodilian trypanosome known to be transmitted by leeches and positioned in the aquatic clade closest to fish trypanosomes. Our data show that caimans can host trypanosomes of the aquatic or terrestrial clade, sometimes simultaneously.

## Highlights

• Species richness and geographical range of caiman trypanosomes.
• *Trypanosoma clandestinus* n. sp. is transmitted by leeches.
• *T. clandestinus* nests in the Aquatic clade closest to fish trypanosomes.
• Caimans were infected by the bite of leeches carrying *T. clandestinus*.

## Graphical abstract

**Figure:**
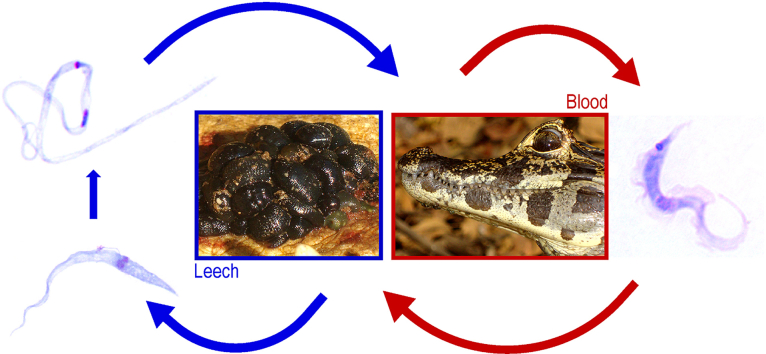

## 1 Introduction

Flagellates of the genus *Trypanosoma* (Euglenozoa, Kinetoplastea, Trypanosomatidae) are obligate parasites of all vertebrate classes and are distributed into two major phylogenetic lineages: the Terrestrial clade composed of trypanosomes of mammals, snakes, lizards, crocodilians and birds and the Aquatic clade containing trypanosomes of aquatic leeches and aquatic (fishes) or semi-aquatic (chelonians, anurans and platypus) hosts and, oddly, a trypanosome of chameleon (Stevens et al., 2001, Hamilton et al., 2005, Hamilton et al., 2007).

Trypanosomes infect Neotropical and Afrotropical crocodilians (Telford, 1995, Telford, 2009, Viola et al., 2008a, Fermino et al., 2013). Crocodilians of the family Alligatoridae predominate in the New World and are distributed in the genera *Caiman*, *Paleosuchus* and *Melanosuchus,* generically called caimans. The Afrotropical Crocodylidae originated in Australasia and after transoceanic dispersal (Oaks, 2011) flourished in South America (Scheyer et al., 2013). In the American continent, Alligatoridae and Crocodylidae encountered plenty of opportunities of trypanosome switching in the wide connected and disconnected wetlands shaping the present day hydrographic basins (Fermino et al., 2013).

Although there are many reports of the occurrence of trypanosomes in reptilians (revised by Telford, 1995, Telford, 2009), only three species from crocodilians were molecularly characterized: *Trypanosoma grayi* (Hoare, 1929, Hoare, 1931), and *Trypanosoma ralphi* and *Trypanosoma terena* (Viola et al., 2008a; Fermino et al., 2013). The reported hosts of these trypanosomes are *Crocodylus niloticus* and *O. tetraspis* in Africa and the caimans *Caiman crocodilus, Caiman yacare* and *Melanosuchus niger* in South America. Available phylogenetic data show very close phylogenetic relationships between the African *T. grayi* from *Crocodylus niloticus* and the Brazilian *T. ralphi* from caimans, as well as between the Brazilian *T. terena* from caimans and an unnamed African trypanosome from *O. tetraspis.* These South American and African crocodilian trypanosomes form the major Crocodilian clade comprising the clades Terena, Ralphi and Grayi; each clade harbors a number of closely related genotypes (Fermino et al., 2013). The phylogenetic relationships between the crocodilian trypanosomes concur with the worldwide transoceanic dispersal of *Crocodylus* during the Miocene that ended in either South America or Africa (Oaks, 2011).

Trypanosomes of the three species discovered in crocodilians to date were detected by culturing, standard PCR and microscopic examination of peripheral blood. However, these methods may fail to detect tissue-dwelling or scarce blood flagellates and culturing could select against trypanosomes that are fastidious or refractory to cultivation. The alligatorid trypanosomes present varied morphology in the blood and tissues of their hosts that could be the result of the inherent polymorphism of trypanosomes or concomitant infections with more than one species (Viola et al., 2008a, Fermino et al., 2013). The alleged polymorphisms of blood trypanosomes may also result from the occurrence of mixed infections that have been disclosed by molecular analyses of parasites directly from blood samples of anurans, fishes and mammals (Ferreira et al., 2007, Gu et al., 2007, Grybchuk-Ieremenko et al., 2014, Thompson et al., 2013, Lemos et al., 2015). In addition, molecular studies revealed a number of trypanosomes from wildlife displaying blood forms that are morphologically indistinguishable (Ferreira et al., 2007, Ferreira et al., 2008, Viola et al., 2008a, Viola et al., 2008b, Viola et al., 2009; Cavazzana et al., 2010; Fermino et al., 2013; Lemos et al., 2015). These facts have prevented the correct appraisal of the whole assemblage of trypanosomes and the extent of their diversity and host and geographical ranges.

Nothing is known about the vectors of trypanosomes of South American caimans. Their close relative *T. grayi* is transmitted to African crocodiles by tsetse flies. A range of hematophagous flies serve as vectors of trypanosomes of reptiles, including tsetse flies for crocodiles (Hoare, 1929, Hoare, 1931) and sand flies for lizards (Ayala, 1971, Ayala and McKay, 1971, Cristensen and Telford, 1972, Viola et al., 2008b). Leeches are known to transmit trypanosomes of aquatic and semi-aquatic animals including fishes (Letch, 1977, Karlsbaak et al., 2005, Khan, 1976, Hayes et al., 2014, Lemos et al., 2015), anurans (Martin and Desser, 1991, Siddall and Desser, 1992), snakes (Pessoa and Fleury, 1969, Chia and Miller, 1984), turtles (Woo, 1969, Siddall and Desser, 1992) and platypus (Paparini et al., 2014).

Trypanosomes of anurans (Ayala, 1971, Martin and Desser, 1991, Siddall and Desser, 1992, Ferreira et al., 2008) and serpents (Chia and Miller, 1984, Viola et al., 2008b) are transmitted by leeches in aquatic environments and by insects such as sand flies and culicids in more terrestrial niches (Desser et al., 1975). Moreover, a survey performed using nested PCR suggested that terrestrial leeches could transmit trypanosomes of toads and marsupials in Australia (Hamilton et al., 2005). Therefore, flies and leeches could transmit the trypanosomes of the semiaquatic caimans, although no vectors have been identified to date.

In the present study, we surveyed for trypanosomes in blood and tissues samples from 122 specimens of South American caimans and the guts of 208 leeches collected from the mouths of caimans. Field surveys, morphological and experimental infections of caimans and phylogenetic analyses were employed to investigate life cycles, species diversity and phylogenetic relationships of crocodilians trypanosomes.

## 2 Materials and methods

### 2.1 Collection sites, caiman handling and blood sampling

**Fig. 1:**
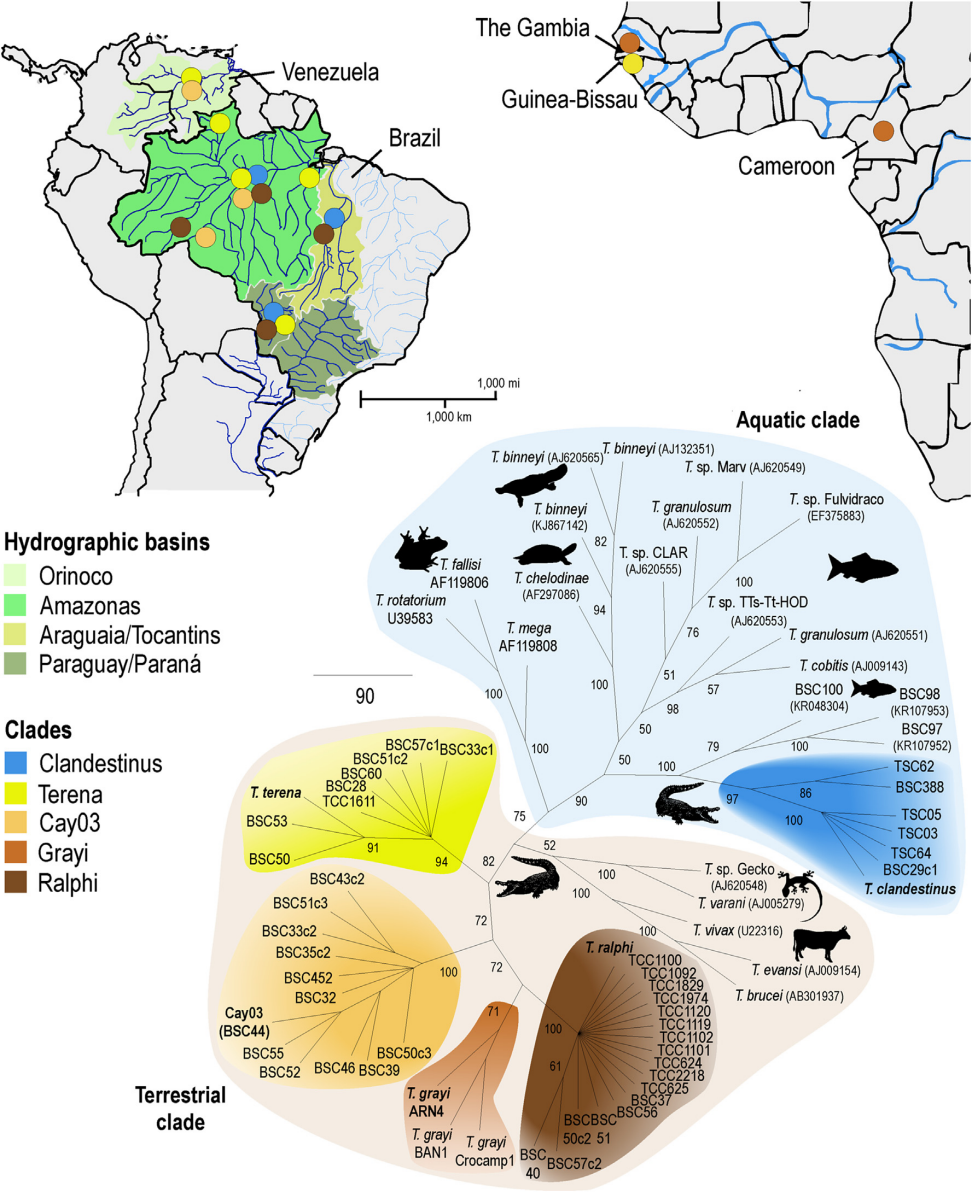
Geographical origin of crocodilian trypanosomes included in the V7V8 SSU rRNA dendrogram inferred to compare the barcode sequences between the new and known trypanosomes from crocodilians and other species of aquatic and semi aquatic hosts. The clade comprising *T. clandestinus* n. sp. nested into the Aquatic clade closely related to fish trypanosomes whereas sequences of the other new species formed the clade Cay03, which clustered with *T. terena*, *T. grayi* and *T. ralphi* in the Crocodilian Terrestrial clade. The host species and geographic origin and Genbank accession numbers of sequences from the crocodilian trypanosomes are shown in Table 1. Numbers at nodes are bootstrap support values >50% (P/ML) derived from 500 replicates.

Caimans were captured in the Amazonian (AM), Araguaia-Tocantins (TO), Paraguay-Paraná (PP) and Orinoco (OR) river basins. The collection sites are shown in Fig. 1 and Table 1. The capture of animals and all ensuing procedures were conducted as described previously (Fermino et al., 2013) according to the recommendations of IBAMA (the Brazilian Institute for the Environment and Renewable Natural Resources), permit Number 10080-2 and the protocol (108/2013) approved by the Committee on the Ethics of Animal Experimentation of the Institute of Biomedical Sciences, University of São Paulo.

Caiman blood samples and liver tissue preserved in ethanol (v/v) were stored in the Blood Sample Collection (BSC) and Tissue Sample Collection (TSC) of the Trypanosomatid Culture Collection of the University of São Paulo (TCC – USP). Total DNA was extracted from blood and tissue samples as previously described using the traditional phenol-chloroform method. DNA samples from all trypanosome-positive crocodilian samples were also preserved in the TCC.

### 2.2 Collection and identification of leeches and survey for trypanosomes

Leeches were collected from the mouths of the caimans, and identified as *Haementeria* sp. by COI barcodes (Folmer et al., 1994). Leeches were immediately preserved in ethanol (v/v) or brought to the laboratory and kept for 15–20 days in plain water until they completely digested the ingested blood meal and became almost transparent. Then, the guts of the leeches were examined by microscopy. Leeches positive for trypanosomes were used for smears on glass slides, inoculation into culture tubes and DNA preparations using the phenol-chloroform method.

### 2.3 PCR amplification and phylogenetic analyses of SSU rRNA and gGAPDH gene sequences

The nested-PCR of SSU rRNA sequences (∼987 bp) of trypanosomes from crocodilian blood samples was performed as described previously (Noyes et al., 1999). PCR amplification of the V7V8 region of the SSU rRNA and gGAPDH sequences from leech trypanosomes was performed as previously described (Borghesan et al., 2013). The nested PCR amplification of gGAPDH sequences was performed using the first round primers (GAPDH SF) 5′ GTG GCG GTK GTY GAC ATG AAC A3′ and (GAPDH SR) 5′ TTG GAG TCR TAG ATR GAG CT3′, and the second round internal primers (GAP 3F) 5′ GTG AAG GCG CAG CGC AAC 3′ and (GAP 5R) 5′ CCG AGG ATG YCC TTC ATG 3′. The two rounds of amplification (30 cycles each) were performed using PCR reaction mixtures and conditions described previously (Borghesan et al., 2013).

Amplified DNA were cloned, and 10–20 clones were sequenced for each gene from each trypanosome sample. Sequencing of cloned amplified DNA allowed for the detection of mixed trypanosome sequences in a single blood sample (Fermino et al., 2013). The SSU rRNA and gGAPDH sequences determined in this study were deposited in GenBank under the accession numbers shown in Table 1.

For phylogenetic inferences using Parsimony (P), Maximum Likelihood (ML) and Bayesian inference (BI) analyses, sequences were aligned using CLUSTALX (Thompson et al., 1997) and manually refined. Three alignments were created: A1, consisting of the V7V8 region of the SSU rRNA gene (687 characters); A2, containing the gGAPDH sequences (608 characters); and A3, consisting of concatenated SSU rRNA and gGAPDH sequences (1.295 characters) using previous alignments including a large set of taxa for guidance (Hamilton et al., 2005). The parsimony and bootstrap analyses were performed using PAUP* version 4.0b10 software (Swofford, 2002) with 500 random sequence addition replicates followed by branch swapping (RAS-TBR). The ML analyses were performed using RAxML v.2.2.3 (Stamatakis, 2006) as described previously (Ferreira et al., 2008, Borghesan et al., 2013). The general time reversible (GTR) model of nucleotide substitution with a proportion of invariable sites and gamma distribution was selected for the gene data sets. The GTR model was used in individual analysis of each gene as well as in the combined Bayesian analysis. The analysis was run for 1,000,000 generations with trees sampled every 100 generations using four chains and 25% of the early sample trees discarded as “burn-in”.

### 2.4 Experimental infection of caimans

Five farm-born *Caiman yacare* 8–10 months old and weighing ∼260 g were kept indoors at 27 °C in large glass tanks of water with a land platform. Blood samples were collected upon the arrival of the animals and for 2 months at ∼15 days intervals to evaluate by nested PCR whether the animals were free of trypanosomes prior to experimental infection. All procedures during the experimental period were performed in accordance with the protocol (#36781-1) approved by the IBAMA and the “Sistema de Autorização e Informação em Biodiversidade” (SISBIO).

Approximately 80 leeches were collected from wild *Caiman yacare* captured in the Miranda river at the PP basin and maintained for 15 days in water at room temperature in the laboratory. Five randomly selected leeches were examined and found to be heavily infected with trypanosomes. The gut contents from these leeches were preserved in ethanol for DNA amplification or smeared onto glass slides for microscopy.

For experimental infections, two caimans had 30 leeches each deposited in their mouths and two additional animals were fed with 30 leeches macerated with meat. One caiman received no leeches and was used as the negative control. Starting on the 7th day after infection, ∼0.5 ml blood samples were collected every ∼15 days for 5 months by caudal venous puncturing. A final blood sample was collected 8 months after the inoculation. Blood samples from each animal were either preserved in ethanol for DNA analysis or immediately inoculated into culture medium as described previously (Fermino et al., 2013) and for the preparation of blood and microhematocrit buffy coat smears on glass slides. The slides were fixed with methanol, stained with Giemsa and examined microscopically. To monitor the experimental infection, DNA preparations from caiman blood were subjected to nested-PCR of V7V8 SSU rRNA and gGAPDH sequences. The amplified products were cloned and 10–20 clones from each sample were sequenced.

## 3 Results

### 3.1 New species disclosed by barcoding of caiman and leech trypanosomes using SSU rRNA and gGAPDH sequences

Preliminary tests showed that the nested PCR methods targeting both SSU rRNA and gGAPDH sequences could detect trypanosomes in the blood of caimans that tested positive or negative by blood microscopic examination and hemoculturing. Then, we examined 122 caiman blood samples and found trypanosome sequences in 40 caimans, yielding a prevalence of ∼33%.

The analyses of V7V8 SSU rRNA and gGAPDH trypanosome sequences obtained from blood or tissue samples from 34 caimans revealed that 23 (68%) of the caimans presented sequences from a single species, whereas 11 (32%) presented mixed infections with two to four trypanosome species; *T. terena* and *T. ralphi* were detected in ∼50% of the mixed infections. Sequences corresponding to two new trypanosome species were detected exclusively using the nested-PCR methods. Samples sharing a new sequence formed a clade (Cay03) that nested together with *T. terena* and *T. ralphi* in the previously described Crocodilian Clade (Fermino et al., 2013); the other new sequences formed the new Clandestinus clade, which were positioned within the Aquatic clade of *Trypanosoma* (Table 1, Fig. 1).

Leeches have not been reported to be vectors of crocodilian trypanosomes, although they are very common and abundant ectoparasites and vectors of other crocodilian parasites (Glassman et al., 1979, Khan et al., 1980, Leslie et al., 2011). We surveyed for trypanosomes in a sample of 208 leeches randomly collected from the mouths of *Caiman yacare* and *Caiman crocodilus;* a total of 120 leeches (∼58%) were found to be positive for trypanosomes by microscopy. The number of leeches (TSC codes) from which trypanosomes were sequenced are listed in Table 1.

The leeches taken from *Caiman yacare* from The Pantanal were identified as *Haementeria* sp. by BLAST analyses of COI sequences (Genbank accession number: leech TSC64Le = KP972452). However, although a single sequence was obtained from 10 randomly selected leeches, the sequences obtained did not completely match any sequence in GenBank, suggesting that they may represent a leech species still lacking a DNA barcode. The leech sequence determined in this study diverged 17% from the closest relative COI sequences from *Haementeria officinalis* (Genbank accession number: JN850907).

**Fig. 2:**
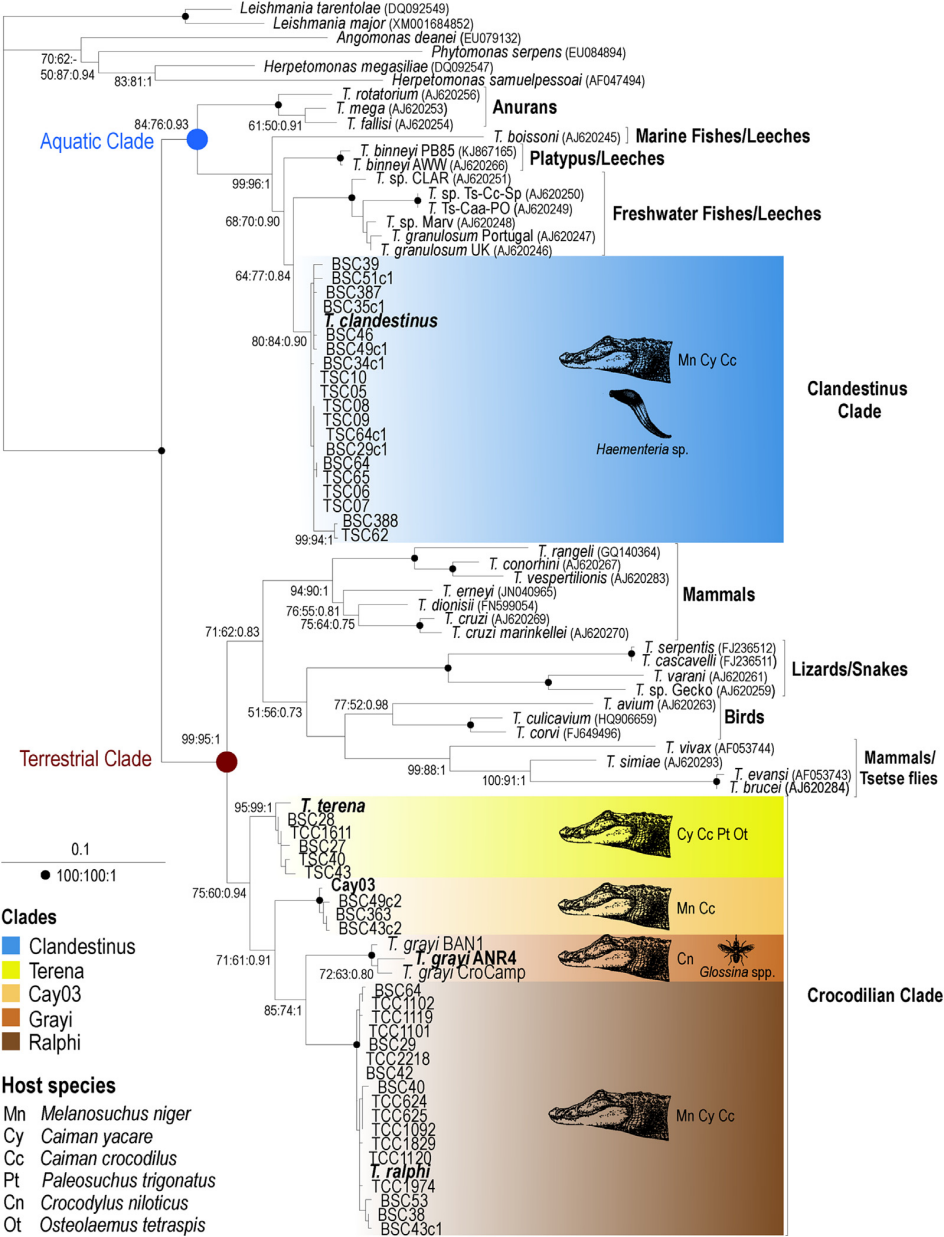
Phylogenetic tree (ML) based on gGAPDH sequences showing the Terrestrial and Aquatic clades of *Trypanosoma* and the positioning of *T. clandestinus*. The Crocodilian clade, which is formed by *T. terena*, *T. ralphi, T. gray* and Cay03 nests in the Terrestrial Clade whereas the Clandestinus clade comprising *T. clandestinus* nests in Aquatic clade. Typanosomatid genera other than *Trypanosoma* were used as outgroups in the phylogenetic trees (608 characters, Ln = −7611.897017). Numbers at nodes are bootstrap support (P/ML) >50% and Bayesian posterior probability >0.25 derived from 500 replicates.

Fresh gut samples of leeches removed from *Caiman yacare* exhibited intense flagellate proliferation. These flagellates always failed to grow when inoculated into the different culture media used to culture a range of trypanosomes including those from the clade Crocodilian (Viola et al., 2008a, Fermino et al., 2013) and anurans (Ferreira et al., 2007). Sequences of V7V8 SSU rRNA and gGAPDH identical to those of the Clandestinus clade from caiman blood samples were the only ones detected in all leeches examined (Table 1, Fig. 1, Fig. 2).

### 3.2 Phylogenetic positioning of caiman and leech trypanosomes within the aquatic clade

In the dendrogram inferred using V7V8 SSU rRNA sequences from trypanosomes of caimans and leeches aligned with sequences of other trypanosome species, the crocodilian trypanosomes of the terrestrial clades Terena, Ralphi and Grayi (Fermino et al., 2013) plus the new clade Cay03 were separated from the Clandestinus clade, which included sequences from trypanosomes allied to fish trypanosomes (Fig. 1). The trypanosomes of the Crocodilian and Clandestinus clades were separated by ∼5.0% V7V8 SSU rRNA divergence.

Inferred phylogenetic trees using gGAPDH sequences (Fig. 2) and concatenated V7V8 SSU rRNA and gGAPDH sequences (Supplementary Material) strongly supported the distribution of the caiman trypanosomes in the Terrestrial and Aquatic clades of *Trypanosoma*. The trypanosomes of the terrestrial Crocodilian clade, comprising *T. terena, T. grayi* and *T. ralphi* plus the new species provisionally referred to as Cay03, clustered with the trypanosomes from lizards, snakes, birds and mammals. The trypanosomes identified in the leeches always clustered with the caiman trypanosomes of clade Clandestinus, comprising highly homogeneous sequences that were strongly supported within the Aquatic clade (Fig. 2). These results enabled the description of the new species *Trypanosoma clandestinus* n. sp. in caimans and leeches.

The divergence of gGAPDH sequences between the five crocodilian trypanosome species ranged from 6.0% to 14%.The largest divergence separated *T. grayi* from *T. clandestinus,* and the shortest divergence separated *T. ralphi* from Cay03. *T. clandestinus* diverged 11.7%, 12.5% and 11% from *T. terena, T. ralphi* and Cay03, respectively. The divergence of gGAPDH sequences within each species varied from 0.5% in *T. clandestinus* to 2.1% in the more heterogeneous clade *T. grayi*. All gGAPDH sequences obtained from leeches (TSC 4–10 and TSC 62–65) were identical to the sequence of *T. clandestinus* (Table 1; Fig. 1, Fig. 2).

Not a single trypanosome sequence of the Crocodilian clade was recovered from leeches. Moreover, *T. clandestinus* was phylogenetically more related to trypanosomes of fishes and placed closest to trypanosomes from Brazilian freshwater fishes than to any other reptilian trypanosome from the aquatic (*Trypanosoma chelodinae*) or terrestrial clade (*Trypanosoma serpentis, Trypanosoma cascavelli, Trypanosoma varani* and *T*. sp. Gecko) (Fig. 1). The terrestrial crocodilian clade was sister to the main clade comprising trypanosomes from snakes, birds, lizards and mammals (Fig. 2).

### 3.3 Leeches as vectors of *Trypanosoma clandestinus* n. sp. and long-term infection of caimans

Although leeches are known to parasitize crocodilians, they had not been shown to transmit their trypanosomes. In this study, we achieved the experimental infection of caimans with trypanosomes from naturally-infected leeches recovered from the mouths of one *Caiman yacare* captured in the Pantanal. SSU rRNA and gGAPDH sequences of the leech trypanosomes corresponded to the sequence previously determined for the leech TSC64 sample (Table 1).

We employed 30 randomly selected leeches for the experimental infestation of two young caimans. After exposure to the infected leeches, we collected blood samples from the caimans at ∼15 day intervals for 5 months. Microhematocrit buffy coats show scarce trypanosomes 45 days after infection. Blood samples tested by PCR confirmed the infection of caimans (BSCs 386 and 387) with *T. clandestinus* (Table 1). The gGAPDH sequences from the infected caiman trypanosome were 99.8–100% similar to those from the leech TSC64. The control caiman and the caimans fed with macerated leeches remained negative by PCR throughout the experiment. Similarly, the ingestion of leeches taken from turtles naturally infected by trypanosomes did not infect turtles (Woo, 1969), suggesting that active leech inoculation of trypanosomes into the bloodstream is required for transmission.

### 3.4 Diversity of crocodilian trypanosomes, mixed infections and lack of host-species restriction

**Fig. 3:**
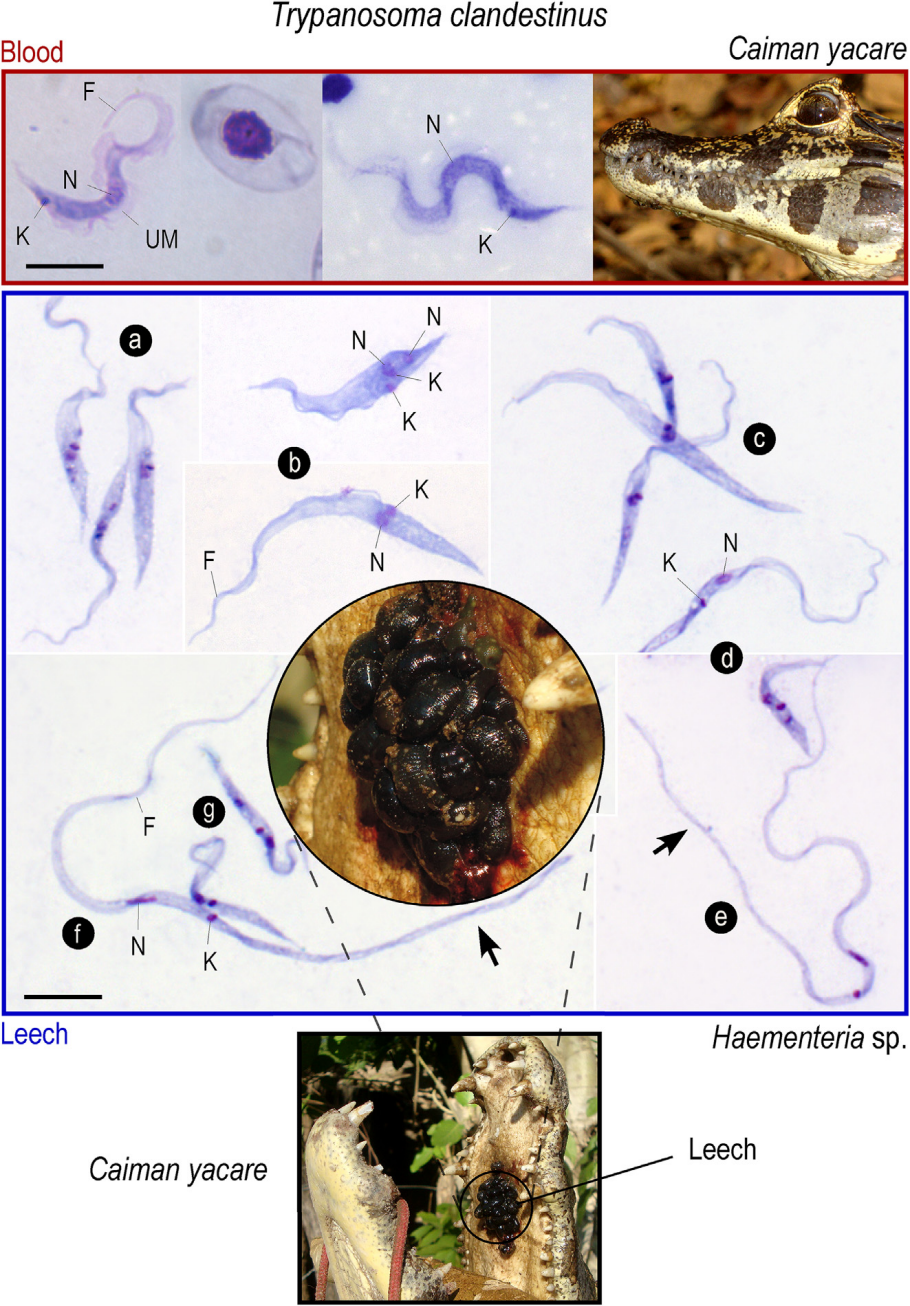
Proposed life cycle of *T. clandestinus* and its developmental and morphological features in caiman blood and leeches. Giemsa-stained blood smears showed blood trypomastigotes of experimentally-infected *Caiman yacare,* and epi- and trypomastigotes found in the gut of one leech of the genus *Haementeria* sp. collected in the mouth of a wild *Cayman yacare* captured in the Pantanal wetland of Brazil. The caiman and the leech trypanosomes were molecularly identified as *T. clandestinus*. (a–c) epimastigotes; (b) epimastigote dividing by binary fission; (d, g) short trypomastigote; (e,f) long and thin trypomastigotes. Arrow points to the long and thin posterior extremity of very long and slender trypomastigotes. K, kinetoplast; N, nucleus; F, flagellum.

The surveys using nested-PCR methods for the detection and barcoding of trypanosomes in the blood of four species of caimans disclosed trypanosome diversity and host-species ranges that are greater than those previously reported by hemoculturing. Two new trypanosome species were uncovered increasing to five the number of species (3) previously known (Fermino et al., 2013). Four species placed into the Terrestrial clades (Terena, Ralphi, Gray and Cay03) and one within the Aquatic clade (Clandestinus) of *Trypanosoma*, each one comprising one trypanosome species and its genotypes (Fig. 1, Fig. 3).

In addition, results corroborated that diverse host species can successfully be used as hosts by different trypanosome species. The *T. clandestinus* clade comprises species found in *Caiman yacare*, *Caiman crocodilus* and *M. niger.* The new clade Cay03 was detected in *M. niger* and *Caiman crocodilus.* The hosts of *T. terena* include four of the six species of Brazilian alligatorids: *M. niger, Caiman crocodilus, Caiman yacare* and *P. trigonatus*. The list of species hosts for *T. ralphi* is similar, except for the absence of *P. trigonatus* (Table 1, Fig. 1, Fig. 2). Thus, among all South American alligatorids, trypanosomes remain unreported only in *Caiman latirostris* and *P. palpebrosus,* which have not been thoroughly examined yet.

Altogether, results from this and previous phylogenetic studies revealed that the species of crocodilian trypanosomes lack host-restriction at species, genus and even family levels. In addition, a crocodilian species may host more than one trypanosome species. Indeed, one-third of the infected caimans harbored two or more trypanosomes. For instance, the *Caiman yacare* blood sample BSC29 yielded trypanosome sequences of the Ralphi and Clandestinus clades. An extreme example is offered by one *M. niger* (BSC51) that carried trypanosome sequences from the four Crocodilian subclades (Table 1, Fig. 1, Fig. 2).

### 3.5 The distribution of trypanosome species in caimans of the South American river basins

This study shows that *T. clandestinus* is widespread in the Amazonian, Araguaia/Tocantins and Parana/Paraguay basins, but was not found in the Orinoco basin (Table 1, Fig. 1). Trypanosomes of clade Cay03 occurred in the Amazonian and Orinoco basins, but were not detected in the other basins including the Parana/Paraguay basin, even though large number of caimans were examined (Table 1, Fig. 1). We have previously shown that *T. terena* and *T. ralphi* were widespread throughout all of the river basins studied (Fermino et al., 2013). Therefore, the analysis of phylogeographical patterns did not support a strong spatial structure for the crocodilian trypanosomes, because most species occurred throughout the geographic distribution range of their hosts (Fig. 1).

### 3.6 Morphology and life cycle of *T. clandestinus* in caimans and leeches

Trypanosomes on blood smears of wild caimans could not be used for description of *T. clandestinus* trypomastigotes due to the common occurrence of mixed infections with two or more trypanosome species. Our source of blood trypomastigotes were the caimans experimentally infected with *T. clandestinus* from leeches harboring exclusively this species of trypanosome. Unfortunately, the caimans presented very low parasitemias rarely detected by microhematocrit, although the infection persisted for at least eight months. The few blood trypomastigotes examined (N = 5) in smears from microhematocrit buffy coats exhibited a serpentine body with a pointed posterior extremity, a central rounded nucleus and a posteriorly located small kinetoplast. The flagellum was short and the undulating membrane was convoluted (Fig. 3). The blood forms of *T. clandestinus* were smaller and thinner than the wider trypomastigotes of *T. terena* and *T. ralphi* (Fermino et al., 2013).

Leeches of the genus *Haementeria* are common ecto-parasites of alligatorids. Because the leeches were rather small the examination of the segments of their digestive tract was not attempted and gut contents were wholly mixed for examination. The leeches removed from the caimans were examined after the engorged blood was completely digested, thus ensuring that the flagellates observed ∼20 days after leeches collection developed in their guts.

As expected for a cyclically transmitted parasite, most leeches were heavily infected with epimastigotes that showed many dividing forms, while some leeches examined after the 20th day presented large numbers of trypomastigotes (Fig. 3). The morphology of the flagellates found in the gut contents of one *Haementeria* leech (TSC09) removed from a *Caiman yacare* that had only sequences (14 clones) identical to that of *T. clandestinus* was selected for the illustration of the trypanosomes found in leeches (defined as type material for species description). The epimastigotes (N = 25) were generally slender, with body lengths varying from 30 to 50 μm (average 42 μm) and widths varying from 2.7 to 4.6 μm (average 3.9 μm). The spherical nuclei and kinetoplasts were close together and located near the middle of the body (Fig. 3a–c) and the flagellum length varied from 2.3 to 6.8 μm (average 5.5 μm). The undulating membrane can be narrow or markedly frilled in the widest forms (Fig. 3b). The polymorphism of the trypomastigotes was much more accentuated than that of the epimastigotes. Short trypomastigotes (N = 15) measuring 23–42 μm in length (average 38 μm) and 2.2 to 2.5 in width (average 2.1 μm) displayed nuclei and kinetoplasts in general displaced towards the anterior end (Fig. 3d, g). Long trypomastigotes, extremely slim at both extremities were abundant. These forms (N = 25) measured 60–107 μm long (average 77 μm) and 0.7–2.5 μm wide (average 1.25 μm) and contained small and elongated nuclei and punctual kinetoplasts located at the center of the body, but not in close proximity to one another. In these thin trypomastigotes the free-flagellum ranged 11–20 μm (average 13.3 μm) (Fig. 3e, f).

Although available data of *T. clandestinus* in leeches and experimentally infected caimans are not sufficient to explain the entire development of this species in its vertebrate host and vector, overall results allowed us to deduce the following life cycle for *T. clandestinus*: *Haementeria* sp. leeches ingest blood containing trypomastigotes upon piercing the caiman buccal mucosa. In the gut of leeches, blood trypomastigotes differentiate into epimastigotes that proliferate and progressively transform into short and long trypomastigotes. Probably, caimans become infected through the bite of leeches carrying trypomastigotes in their proboscis. Although few trypomastigotes were found in the peripheral blood of infected caimans, each leech ingested a considerable volume of caiman blood yielding the high prevalence of *T. clandestinus* in leeches.

## 4 Taxonomic summary

### 4.1 New species description

Phylum Euglenozoa Cavalier-Smith 1981; Class Kinetoplastea Honigberg 1963; Order Trypanosomatida (Kent 1880) Hollande 1982. Genus *Trypanosoma* Gruby 1843. *Trypanosoma clandestinus* Teixeira and Camargo n. sp.

**Type Material:** Hapantotype: trypomastigotes on glass slides of smears of blood sample BSC386 from *Caiman yacare,* and epi- and trypomastigotes on smears of gut contents from the leech *Haementeria* sp. (sample TSC 64). The slides are deposited at the Trypanosomatid Culture Collection of the University of São Paulo (TCC–USP), Brazil. Leeches infected with *T. clandestinus* are cryopreserved in liquid nitrogen. Paratypes: trypomastigotes on glass-slide blood smears of *Caiman yacare* blood sample BSC387 stored at TCC-USP. **Vertebrate type host**: *Caiman yacare* (Crocodylia, Alligatoridae). Vertebrate additional hosts: *Caiman crocodilus* and *Melanosuchus niger.* **Invertebrate type host:** *Haementeria* sp. (Hirudinea, Rhynchobdellida), identified by COI sequences deposited in GenBank (KP972452). **Habitats**: blood of caimans and digestive tube of leeches. **Type locality**: Miranda River (S20° 14′ W56° 22′), Mato Grosso do Sul, the Pantanal, Brazil. **Additional localities**: AM and AT river basins (Table 1). **Morphology:** Caiman blood trypomastigotes and leech epi- and trypomastigotes shown in Fig. 3. **Molecular diagnosis:** DNA sequences deposited in GenBank of trypanosomes from *Caiman yacare* (BSC386) of the genes V7V8 SSU rRNA (KP768285) and gGAPDH (KP768260) and from trypanosomes of the leech *Haementeria* sp. (TSC64), V7V8 SSU rRNA (KR107951) and gGAPDH (KP768270). **Etymology**: clandestinus, Lat. n. m.: hidden, concealed, occult.

**Taxonomical Comments:** The lack of host specificity of caiman trypanosomes and the existence of similar blood trypomastigotes shared by different species, intra-species polymorphisms and mixed infections do not permit the assumption that the trypanosomes observed in blood smears belong to a single or multiple species. Therefore, blood trypomastigotes from naturally infected caimans could not be the type material, unless they are proven to belong to a single species. In the absence of derived cultures, it is necessary to verify whether only one trypanosome sequence is present through sensitive PCR and sequencing of a large number of cloned sequences from each blood sample. Alternatively, flagellates from vectors (epi- and trypomastigotes) exhibiting sequences of a single trypanosome species and blood trypomastigotes from animals experimentally infected with molecularly authenticated trypanosomes from the vectors can serve as type material. In this paper, we used the last two procedures to validate the new species because cultures of *T. clandestinus* are not available.

In addition, sequence divergences separating *T. terena, T. ralphi* and Cay03 were large enough to permit considering Cay03 as a new species within the Terrestrial clade. However, we still do not have a proper type material to taxonomically validate Cay03 as a new species.

## 5 Discussion

Recent molecular studies have uncovered a vast diversity of wildlife parasites within the genus *Trypanosoma*. However, the molecular phylogenetic studies of trypanosomes of reptiles are still limited to a few species of turtles (Jakes et al., 2001), lizards and snakes (Hamilton et al., 2007, Viola et al., 2008b, Viola et al., 2009) and crocodilians (Viola et al., 2008a, Fermino et al., 2013). In this study, surveys of trypanosomes in blood samples from South American caimans using sensitive nested-PCR methods revealed an unsuspected high prevalence (33%) of infection and common mixed infections by two or more trypanosome species (45%). The phylogenetic positioning of caiman trypanosomes using SSU rRNA and gGAPDH sequences increased our knowledge about the species richness of crocodilian trypanosomes and raised the number of molecularly characterized species from three to five. Two phylogenetically-supported new trypanosome species were identified. One species was designated *T. clandestinus* n. sp. and molecularly and morphologically characterized in the present study. This species was shown to be transmitted by leeches and to cluster within the Aquatic clade of *Trypanosoma*. Altogether, the information concerning the development and morphological features in leeches and in blood of caimans experimentally infected with this trypanosome species through the bite of leeches, permitted us to outline the probable life cycle of *T. clandestinus.*

Due to the frequent occurrence of mixed infections and the large range of host species, it is impossible to identify a new species of crocodilian trypanosome based on the morphology of blood trypanosomes and host of origin. Phylogenetic analyses are obligatory and, preferably, should be preceded by culturing and morphological characterization of the candidate new species. Before this study, the use of these combined approaches prompted the description of two species of crocodilian trypanosomes (*T. terena* and *T. ralphi*) in South American alligatorids (Fermino et al., 2013). Similarly, cultures derived from tsetse flies and *C. niloticus* allowed the molecular characterization of *T. grayi* (Stevens et al., 2001, Hamilton et al., 2009, Fermino et al., 2013).

In contrast with *T. terena, T. ralphi* and *T. grayi,* which are commonly detected by microscopy, hemocultures and conventional PCR, *T. clandestinus* remains hidden in its crocodilian host and is exclusively detected by highly sensitive nested PCR methods. Contrasting with the very low parasitemia of caiman hosts, *T. clandestinus* can be easily detected by microscopy and conventional PCR in leeches of the genus *Haementeria*, which are ectoparasites often found in large clumps in the buccal cavity of crocodilians. The abundant flagellates multiplying in the digestive tract of *Haementeria* sp. taken from caimans did not grow in culture under any of the conditions we attempted but offered sufficient material for morphological and molecular analyses, and for experimental infections of caimans*. T. clandestinus*-carrying leeches were used to infect farm-raised *Caiman yacare*. Blood trypanosomes from the infected caimans yielded V7V8 SSU rRNA and gGAPDH sequences identical to those determined for the trypanosomes multiplying in leeches. Thus, results demonstrated that leeches were capable of transmitting *T. clandestinus* to *Caiman yacare*.

*T. clandestinus* is the first crocodilian trypanosome known to be transmitted by leeches and placed into the Aquatic clade. This finding is in agreement with the role of aquatic leeches as vectors of most trypanosomes of fishes, anurans, turtles and the platypus (Martin and Desser, 1991, Siddall and Desser, 1992, Hayes et al., 2014, Paparini et al., 2014). *T. clandestinus* was the sole trypanosome identified in the gut of *Haementeria* sp., even in leeches taken from caimans infected with other two or more trypanosome species. This finding support our hypothesis that the crocodilian trypanosomes of the Terrestrial clade are transmitted by hematophagous flies, similarly to some trypanosomes of anurans, lizards and snakes (Ayala, 1971, Ayala and McKay, 1971, Ferreira et al., 2008, Martin and Desser, 1991, Siddall and Desser, 1992, Viola et al., 2008b, Viola et al., 2009) and *T. grayi* of African crocodiles (Hoare, 1929, Hoare, 1931).

In this study, phylogenetic analyses evidenced, for the first time, that crocodilians of distinct species harbor phylogenetically distant trypanosomes of the two major *Trypanosoma* clades. The Aquatic clade harbors *T. clandestinus* transmitted by leeches and highly phylogenetically related to fish trypanosomes. The Terrestrial clade harbors *T. grayi* transmitted by tsetse flies plus *T. terena*, *T. ralphi* and Cay03 of unknown vectors. In contrast, all anuran trypanosomes nested in the Aquatic clade even though they are transmitted by leeches or by flies (Ferreira et al., 2007, Ferreira et al., 2008). It will be interesting to see whether trypanosomes from aquatic snakes transmitted by aquatic leeches (Pessoa and Fleury, 1969, Chia and Miller, 1984) cluster in the Aquatic clade or in the Terrestrial lizard-snake clade of trypanosomes transmitted by sand flies (Viola et al., 2009). To date, a chameleon trypanosome of unknown vector is the only trypanosome from lizard placed in the Aquatic clade (Stevens et al., 2001, Hamilton et al., 2007).

Here, we demonstrated that different trypanosome species co-infect different species of crocodilians across South American river basins. The identification of new hosts and new trypanosomes permits us to anticipate that the species richness of crocodilian trypanosomes is much larger than presently known. However, the task of effectively cataloguing the species richness will have to face not only the conceptual issue of species delimitation (Adams, 2001, De Queiroz, 2007), but also the operational problems of new species descriptions. Collecting blood samples and leeches from wild crocodilians in their natural habitats can be very difficult. Moreover, the existence of species such as *T. terena* and *T. ralphi* that share morphologically indistinguishable blood and culture forms (Viola et al., 2008a, Fermino et al., 2013) or non-culturable species as *T. clandestinus* adds difficulties to the appreciation of the trypanosome diversity. *T. clandestinus* does not belong to the cryptic category of parasites (i.e., two or more species morphologically perceived as a single species), but to a category of parasites that go unnoticed in host blood samples by common methods of detection/identification of trypanosomes. Any inventory of parasite biodiversity and the proposition of well-supported hypotheses about their evolutionary histories depends on the sensitive detection and correct identification of species. The enormous realm of wildlife parasites remains largely underexplored and the occurrence of cryptic, clandestine and misclassified parasite species further complicates the prospect of such an inventory (Criscione et al., 2005, Bickford et al., 2007, De León and Nadler, 2010; Detwiler et al., 2010; Nadler and De León, 2011, Poulin et al., 2011b, Bray and Cribb, 2015). Further studies are required to assess whether the specificity of the crocodilian trypanosomes is restricted to closely related crocodilian species or whether they can exploit distantly related hosts of other taxa across their broad geographic range (Poulin et al., 2011a). Caimans are known to be generalist predators that feed on diverse preys including fish, turtles, anurans and other aquatic and terrestrial animals. Animals sharing ecological niches and serving as food are known to share parasites with crocodilians (Junker and Boomker, 2006, Tellez and Nifong, 2014). In this context, it is worth emphasizing that *T. clandestinus* was placed closest to fish trypanosomes within a major assemblage of parasites transmitted by aquatic leeches. It is known that fishes and anurans play a role of paratenic hosts of *Hepatozoon caimani,* a highly prevalent caiman hemoparasite transmitted by culicids (Viana et al., 2012, Pereira et al., 2014). Therefore, it is possible that fish and other aquatic vertebrates share trypanosomes with caimans, including *T. clandestinus*, due to host-switching mediated by aquatic leeches and predation by crocodilians. We are currently examining trypanosomes of fishes and anurans that serve as food for the caimans in the Pantanal to investigate the role of ecological fitting favoring host-switching of trypanosomes sharing ecological niches. So far, we have not found trypanosomes identical to *T. clandestinus* in anurans (Ferreira et al., 2007) sharing ponds with *Caiman yacare.* However, preliminary phylogenetic analysis revealed partial SSU rRNA sequences closely related to *T. clandestinus*, but not identical, in blood samples of fishes (BSCs 97, 98, 100) from The Pantanal (Fig. 1) and other Brazilian hidrographic basin (Lemos et al., 2015).

In addition, misclassified trypanosome species may obscure the etiology of infections of wild and farmed caimans. Understanding vectors and parasite species-specific associations have implications for conservation, management and production purposes. Molecular approaches have only recently been used to investigate helminthiasis of endangered species or farmed crocodilians (Zhao et al., 2015, Lott et al., 2015). There are no studies addressing the pathological effects of trypanosome infections in caimans. We did not find any obvious sign of illness in the caimans naturally and chronically infected (8 months) with *T. clandestinus*. However, the demonstration of trypanosomes in blood and tissue imprints of kidney and lung of wild crocodilians (Lainson, 1977, Viola et al., 2008a) deserve further investigations. Trypanosomes in general have been considered non-pathogenic in wildlife, although studies are scarce and in general superficial. Recently, studies about trypanosomes infecting endangered Neotropical primates and Australian marsupials started producing relevant data concerning wildlife health and conservation (Maia da Silva et al., 2008, Thompson et al., 2013, Botero et al., 2013, Austen et al., 2015).

Results from the present study disclosed an increasing species richness, enlarged host/parasite geographical range, aquatic and terrestrial cycles of transmission and new vectors (leeches) of crocodilian trypanosomes. The diversity and phylogenetic relationships of these trypanosomes likely have been shaped by transoceanic dispersion and ecological fitting with multiple events of host-switching during the long shared evolutionary histories of trypanosomes, crocodilians of different families and leeches.

## Figures and Tables

**Table 1:** Sequences of gGAPDH and V7V8 SSU rRNA trypanosomes from caimans and leeches from South American river basins.

Sample^#^	Host	River basin	GPS	GenBank Accession numbers	
gGAPDH	V7V8 SSU rRNA
Clade clandestinus					
*T. clandestinus* (BSC386)	*C. yacare*	PP	19°57′S 57°01′W	KP768260	KP768285
*T. clandestinus* (TSC64)	*Haementeria*	PP	19°57′S 57°01′W	KP768270	KR107951
BSC29c1*	*C. yacare*	PP	19°57′S 57°01′W	KP768252	KP768284
BSC34c1*, BSC35c1*, BSC39, BSC46, BSC49c1*, BSC51c1*	*M. niger*	AM	2°19′S 65°70′W	KP768253-8	–
BSC64c1*	*C. crocodilus*	AT	7°32′S 49°22′W	KP768259	–
BSC387	*C. yacare*	PP	19°57′S 57°01′W	KP768261	–
BSC388	*C. crocodilus*	AT	7°66′S 49°29W	KP768262	KP768286
TSC03	*Haementeria*	PP	19°57′S 57°01′W	–	KP768287
TSC05	*Haementeria*	PP	19°57′S 57°01′W	KP768263	KP768288
TSC06, TSC07, TSC08, TSC09, TSC10	*Haementeria*	PP	19°57′S 57°01′W	KP768264-8	–
TSC62	*Haementeria*	AT	7°66′S 49°29W	KP768269	KP768289
TSC65	*Haementeria*	PP	19°57′S 57°01′W	KP768271	–
Clade terena					
***T. terena*** (TCC621)	*C. yacare*	PP	19°57′S 57°01′W	EU596252	EU596256
BSC27	*O. tetraspis*^*#*^	GB	11°60′N 15°04′W	KF546505	–
BSC28	*C. crocodilus*	OR	6°83′N 67°69′W	KF546504	KF546518
BSC33c1*, BSC51c2*, BSC57c1*	*M. niger*	AM	2°19′S 65°70′W	–	KP768290-2
BSC50*, BSC53	*M. niger*	AM	2°19′S 65°70′W	–	KF546519-20
TCC1611	*C. yacare*	PP	19°57′S 57°01′W	KF546517	KF546503
BSC60	*C. crocodilus*	AM	7°27′S 64°80W	–	KP768293
TSC40	*P. trigonatus*	AM	3°80′N 61°73′W	KP768272	–
TSC43	*C. crocodilus*	AM	3°20′S 51°86′W	KP768273	–
Clade ralphi					
***T. ralphi*** (TCC1838)	*M. niger*	AM	7°27′S 64°80W	KF546512	KF546527
BSC29*	*C. yacare*	PP	19°57′S 57°01′W	KF546515	–
BSC37	*M. niger*	AM	2°19′S 65°70′W	–	KP768294
BSC38	*M. niger*	AM	2°19′S 65°70′W	KP768274	–
BSC40	*M. niger*	AM	2°19′S 65°70′W	KP768275	KP768295
BSC42, BSC43c1*	*M. niger*	AM	2°19′S 65°70′W	KP768276-7	–
BSC50c2*	*M. niger*	AM	2°19′S 65°70′W	–	KP768296
BSC51*	*M. niger*	AM	2°19′S 65°70′W	–	KF546524
BSC53	*M. niger*	AM	2°19′S 65°70′W	KP768278	–
BSC56	*M. niger*	AM	2°19′S 65°70′W	–	KF546525
BSC57c2*	*M. niger*	AM	2°19′S 65°70′W	–	KP768297
BSC64c2*	*C. crocodilus*	AT	7°32′S 49°22′W	KF546516	–
TCC624	*C. yacare*	PP	19°57′S 57°01′W	EU596253	EU596257
TCC625, TCC1100	*C. yacare*	PP	19°57′S 57°01′W	KF546506	EU596259
TCC1092	*C. yacare*	PP	19°57′S 57°01′W	EU596258	EU596254
TCC1101, TCC1102, TCC1119	*C. yacare*	PP	19°57′S 57°01′W	KF546507-9	EU596261-3
TCC1120	*C. yacare*	PP	19°57′S 57°01′W	KF546510	EU596255
TCC1829	*C. crocodilus*	AM	7°27′S 64°80W	KF546511	KF546521
TCC1974	*C. yacare*	PP	19°57′S 57°01′W	KF546513	KF546522
TCC2218	*C. crocodilus*	AT	7°32′S 49°22′W	KF546514	KF546523
Clade Cay03					
BSC32, BSC33c2*, BSC35C2*, BSC39	*M. niger*	AM	2°19′S 65°70′W	–	KP768298-301
BSC43c2*, BSC44,	*M. niger*	AM	2°19′S 65°70′W	KP768279-80	KP768302-3
BSC46	*M. niger*	AM	2°19′S 65°70′W	–	KP768304
BSC49c2*	*M. niger*	AM	2°19′S 65°70′W	KP768281	–
BSC50c3*, BSC51c4*, BSC52	*M. niger*	AM	2°19′S 65°70′W	–	KP768305-7
BSC55	*M. niger*	AM	2°19′S 65°70′W	KP768282	KP768308
BSC363	*C. crocodilus*	AM	8°80′S 63°95′W	KP768283	–
BSC452	*C. crocodilus*	OR	6°83′N 67°69′W	–	KR107954

# Identification code of the samples deposited at the Trypanosomatid Culture Collection of the University of São Paulo (TCC-USP): TCC, culture number; BSC, blood sample number; TSC, tissue sample number. *Mixed infections, “c” designates the clone number. South American river basins: AM, Amazonas; AT, Araguaia-Tocantins; PP, Paraná-Paraguay; OR Orinoco. # GB, Guinea Bissau.

## Appendix A Supplementary data

Fig. S1 Phylogenetic tree (ML) based on concatenated V7V8 SSU rRNA and gGAPDH sequences of *T. clandestinus* and other trypanosomes of the Aquatic and Terrestrial clades (GenBank accession numbers are in Table 1). Trypanosomatid genera other than *Trypanosoma* were used as outgroups in the phylogenetic trees (1.178 characters, Ln = −17937.481223). Numbers at nodes (P/ML/BI) are bootstrap support values >50% and Bayesian posterior probability >0.25, derived from 500 replicates.
