# Supplementary figures and images for: Field and experimental evidence of a new caiman trypanosome species closely phylogenetically related to fish trypanosomes and transmitted by leeches
Source: Int J Parasitol Parasites Wildl. 2015 Oct 21;4(3):368–78. doi: 10.1016/j.ijppaw.2015.10.005 (PMC4683569; doi:10.1016/j.ijppaw.2015.10.005)

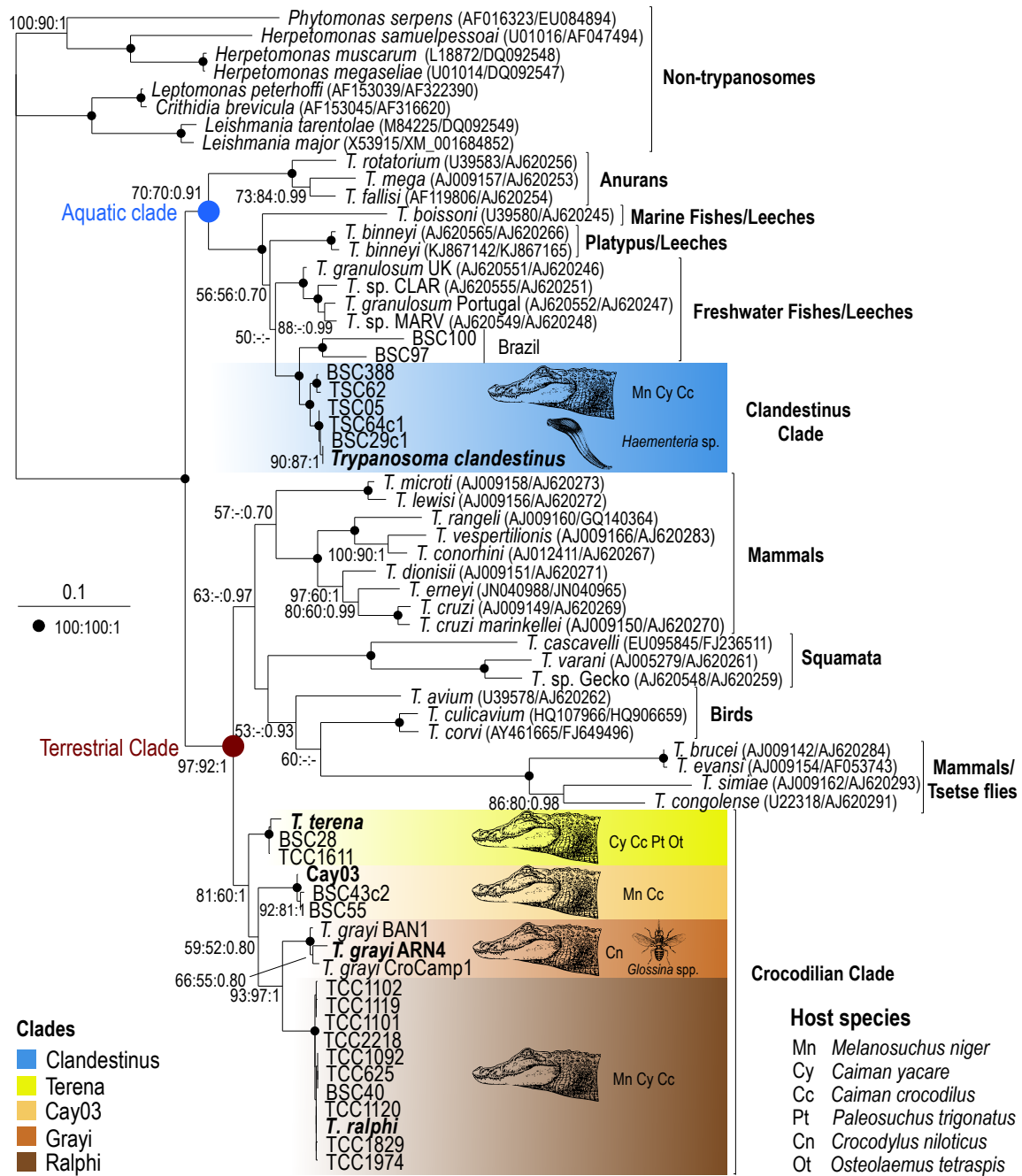

Supplement: Fig. S1 — Phylogenetic tree (ML) based on concatenated V7V8 SSU rRNA and gGAPDH sequences of T. clandestinus and other trypanosomes of the Aquatic and Terrestrial clades (GenBank accession numbers are in Table 1). Trypanosomatid genera other than Trypanosoma were used as outgroups in the phylogenetic trees (1.178 characters, Ln = −17937.481223). Numbers at nodes (P/ML/BI) are bootstrap support values >50% and Bayesian posterior probability >0.25, derived from 500 replicates. [file mmc1.pdf]
